# Declining trends in conception rates in recent birth cohorts of native Danish women: a possible role of deteriorating male reproductive health

**DOI:** 10.1111/j.1365-2605.2007.00827.x

**Published:** 2008-04

**Authors:** Tina Kold Jensen, Tomáš Sobotka, Martin A Hansen, Anette Tønnes Pedersen, Wolfgang Lutz, Niels E Skakkebæk

**Affiliations:** * Department of Growth and ReproductionRigshospitalet, Copenhagen, Denmark; † Department of Environmental Medicine, University of Southern DenmarkOdense, Denmark; ‡ Vienna Institute of DemographyVienna, Austria; § Department of GynaecologyRigshospitalet, Copenhagen, Denmark; ¶ International Institute for Applied Systems Analysis (IIASA)Laxenburg, Austria

**Keywords:** fertility rate, male reproductive health, total natural conception rate

## Abstract

Recent findings of poor semen quality among at least 20% of normal young men in Denmark prompted us to use unique Danish registers on births and induced abortions to evaluate a possible effect of the poor male fecundity on pregnancy rates among their presumed partners – the younger cohorts of women. We have analysed data from the Danish birth and abortion registries as well as the Danish registry for assisted reproduction (ART) and defined a total natural conception rate (TNCR), which is equal to fertility rate plus induced abortion rate minus ART conception rate. A unique personal identification number allowed the linkage of these databases. Our database included 706 270 native Danish women born between 1960 and 1980. We used projections to estimate the fertility of the later cohorts of women who had not yet finished their reproduction. We found that younger cohorts had progressively lower TNCR and that in terms of their total fertility rate, the declining TNCR is compensated by an increasing use of ART. Our hypothesis of an ongoing birth cohort-related decline in fecundity was also supported by our finding of increasing and substantial use of ART in the management of infertility of relatively young couples in the later cohorts. Furthermore, the lower rates of induced abortion among the younger birth cohorts, often viewed as a success of health education programs, may not be fully explained by improved use of contraception. It seems more likely that decreased fecundity because of widespread poor semen quality among younger cohorts of otherwise normal men may explain some of the observed decline in conception rates. This may imply increasing reproductive health problems and lower fertility in the future, which is difficult to reverse in the short term. The current and projected widespread use of ART in Denmark may be a sign of such an emerging public health problem.

## Introduction

A widespread decline in semen quality among men in the Western world has been reported during the past decades. A previous study from our group (Carlsen *et al.*, 1992) on apparent declining semen quality caused controversy (Jouannet *et al.*, 2001) and prompted Swan (Swan *et al.*, 2000) to carry out an updated and expanded meta-analysis, which confirmed a trend towards lower sperm counts in Europe as well as in the United States. Subsequently, Scottish researchers (Irvine *et al.*, 1996) could confirm the French findings (Jouannet *et al.*, 2001) and relate a decline in sperm counts to a birth cohort phenomenon, whereby men born more recently had lower semen quality. Also, a re-analysis of historic Danish data showed a similar birth cohort trend (Bonde *et al.*, 1998b; Andersen *et al.*, 2000). Previous studies on trends in semen quality were limited by the fact that they were based on retrospective data collected for other purposes.

These facts inspired us and others to perform prospective studies on semen quality of several thousand men aged between 18 and 20 years from the general population and among partners of pregnant women, usually men in their 30s. These studies, which included men from the Nordic countries, Germany, France, UK and the Baltic countries, as well as from Japan and the USA, have shown significant geographic differences in semen quality (Jorgensen *et al.*, 2001, 2002; Swan *et al.*, 2003). They have also clearly shown that men of younger birth cohorts (born in the 1970s) have significantly poorer semen quality than men born in previous decades. Particularly the young Danish men had alarmingly low semen quality (Andersen *et al.*, 2000; Jørgensen *et al.*, 2001, 2002; Punab *et al.*, 2002).

However, as an ejaculate of a fertile man most often contains 40–300 million sperms, fecundity and fertility may be unaffected by a reduction in sperm concentration, until a certain lower threshold is reached. Several studies now show that around 20% of young men may have reached that threshold (Jorgensen *et al.*, 2002). The recent data from surveillance of semen quality (our ongoing project for the Danish Ministry of Health) shows that both sperm numbers and sperm morphology are very poor among some otherwise healthy young Danish men (Jørgensen *et al.*, 2006): as a matter of fact 21% Danish young men had sperm counts below 20 mill/mL (lower WHO limit of normal sperm concentration) and 43% of them below 40 mill/mL (Andersen *et al.*, 2000). Importantly, using modern rigorous techniques for analysis of morphology of sperm, it was shown that the average young man from the general population did not have more than 7% normal sperms. This number should be seen in light of other studies which have demonstrated that a high proportion of men in whom the percentages of morphological normal sperms are below 5% are subfecund (Guzick *et al.*, 2001). We therefore believe that we may now have reached a level where semen quality of a significant segment of men in the population is so poor that it may contribute to the current widespread use of assisted reproduction (ART) (Andersen & Erb, 2006) and particularly to the increasing trends in intracytoplasmic sperm injection (ICSI) treatments because of male infertility (Andersen & Erb, 2006). In 2004, 6% of Danish newborns were conceived through ART when intrauterine insemination (IUI) was included. It therefore seems important to consider the possibility that decreased fecundity (ability to conceive) may also contribute to the decreasing fertility rate.

Prompted by the findings of semen quality in the subfecund range in at least 20% and possibly as many as 40% of young men, we decided to use unique Danish registers on births and abortions to evaluate a possible effect of the poor male semen quality on pregnancy rates among their presumed partners – the younger cohorts of women. We have been able to reconstruct the cohort fertility rates, cohort induced abortion rates as well as conceptions after the use of ART [specifically, in vitro fertilization (IVF) and ICSI] and thereby also the cohort trends in the ‘total natural conception rate’ (TNCR). Cohort format of data is more appropriate for the purpose of our study, because poor semen quality is hypothesised to affect especially younger cohorts of Danish men (Andersen *et al.*, 2000; Jorgensen *et al.*, 2002). In order to obtain a relatively homogeneous population, we have limited our investigation to native Danish women and excluded the subpopulation of immigrants who may have different reproductive behaviour. As the cohorts were born from 1960 to 1980 and not all have yet completely finished their reproductive activity we also calculated and projected their future fertility, induced abortion rates and the ART conception rates.

## Materials and methods

### Definitions

In order to evaluate trends in the number of pregnancies obtained without ART rather than the number of births, we studied the TNCR, defined here as: 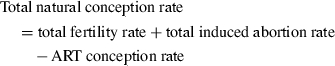 where ART represents assisted reproductive techniques, which included IVF and ICSI (and excluded IUI because of incomplete data).

### Registers

In Denmark, all citizens are registered with a unique personal identification number from the Danish Civil Registration System. We used the following five registers and data sources: The Medical Danish Birth Registry, The Registry of Legally Induced Abortions, National Patient Registry, The Danish IVF Registry and Statistics Denmark. For each entry in each of these registries, the CPR-number is recorded which allows a unique identification of all registered persons and thus also linking of data from different registries. From The Medical Birth Registry and The Registry of Legally Induced Abortions information about all births (live and stillbirths) and legally induced abortions with CPR number of mothers from 1973 to 1994 was obtained. From 1995, the National Patient Registry took over the registration from both registries and information was obtained through this registry. We obtained permission to link and store these data from the Danish Data Protection Agency.

We linked data from the Medical Birth Registry with the register of resident population in Denmark, provided by Statistics Denmark. We included in our analysis all women who had Danish parents, were born in Denmark between 1960 and 1980 and were resident in Denmark as of the first of January 2004. This eliminated the possible impact of migrants on the cohort fertility trends. In total, our records contained 706 270 women of whom 459 838 (65%) had become mothers by the end of 2003. We linked births and population data with the data on ART treatments, provided by the National Board of Health. These data, covering ART treatments since 1994, did not enable us to reconstruct a complete history of ART cycles for Danish women born after 1960. First, some women born in the early 1960s received infertility treatment before 1994 and were not registered in the database. Thus, our analysis slightly underestimated the proportion of ART births and the impact of ART on fertility among women born until the mid-1960s. Second, we only had records on the first initiation of an ART cycle for each woman who had received this treatment between 1994 and 2003 and achieved pregnancy. In our analysis, we assumed that all births of a woman ever undergoing an ART treatment resulted from an ART if they occurred after the initiation of her first ART cycle. This assumption lead to a slight overestimation of ART births in our analysis, as some women achieved ‘natural’ conception through sexual intercourse after they had used ART. Third, our analysis excluded IUI and frozen embryo replacements (FER). Whereas FER accounted only for 0.3% of all children born in Denmark in 2002, IUI are estimated to account for 2.0% of all births (Andersen & Erb, 2006). In contrast with the ART treatments, it is not mandatory to report IUI treatments and their registration is therefore not complete. In addition, IUI treatments are frequently provided in private medical establishments. Data on IUI treatments were therefore not included in our analysis.

We obtained ethical permission from the ethical board under the National Board of Health to merge the data and the personal identification numbers were anonymized and kept separate from the other data.

### Cohort projections of fertility, abortion and ART fertility

We formulated three projection scenarios of fertility, two scenarios of induced abortion rates, and two scenarios of the proportion of ART on fertility rates by age, which reflect different assumptions about the continuation of the shift towards delayed childbearing and the extent of ‘recovery’ of delayed fertility at higher reproductive ages [except for abortions, these projections are described in detail in another study (Sobotka *et al.*, 2007)]. Here we concentrated only on the results of scenarios that we considered the most likely. These scenarios assumed that the trends observed in the late 1990s and early 2000s will continue for a specified period into the future.

The trend scenario of fertility rates (Sobotka *et al.*, 2007) assumed that the trends in age and parity-specific childbearing probabilities observed during 1998–2003 will continue for another 10 years (i.e., until 2013) and, subsequently, these probabilities remain constant. A linear extrapolation has been used in this projection. An identical methodology was employed for projecting trends in abortion rates, except for the missing specification of abortion by order: the trend scenario of abortion rates used here assumed that age-specific trends in abortion rates during 1998–2003 will continue until 2013 and, subsequently, abortion rates remain constant.

With respect to ART fertility rates, we focused on the second scenario (Sobotka *et al.*, 2007), which assumed that the age-specific proportion of ART-fertility (specified for single years of age, distinguishing between first births and higher-order births) remained fixed at the level observed during 2000–2003 until 2007. Subsequently, it changes from 2008 onwards in line with the trend observed during 1998–2003. Because the ART use in some age categories fluctuated considerably, we decided to make this simple one-time change extrapolation rather than to formulate a projection with annual changes in ART-fertility. This scenario does not assume any further increase in the age-specific proportion of ART fertility after 2008. By selecting this rather ‘conservative’ scenario, we took into account uncertainty about future trends in ART use. It is possible that the demand for ART in Denmark may become saturated (not increasing further) in the near future and therefore our scenario does not envision a further rate of increase in the ART share of age-specific fertility rate.

## Results

**Figure 1:**
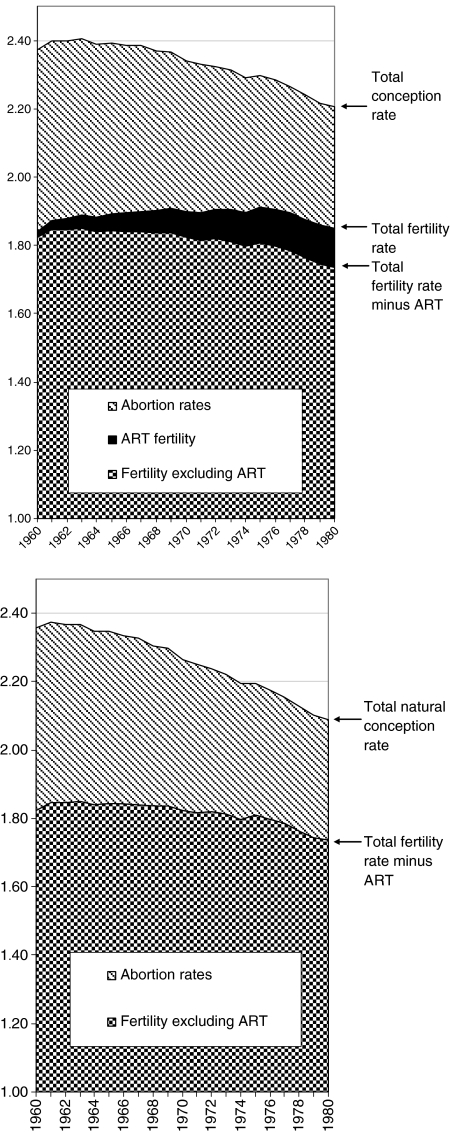
The total conception rate, the total induced abortion rates, and the total fertility rate with and without assisted reproduction (ART) among birth cohorts of native Danish women born in 1960–80. Note: The scale has been magnified. Rates are based on observed data for the period through 2003 and trend projections for the subsequent years.

Figure 1 shows the total conception rate, which includes induced abortions, total fertility rate (TFR) and ART fertility rates (included in left-hand panel only). It is important to bear in mind that whereas the 1960 cohort had almost exhausted its reproductive phase by 2004 (starting year of the projection) and only a small fraction of births, abortions and ART births have been projected for this cohort, for the cohorts born from 1974 onwards, more than half of their births and over 40% of their induced abortions had not occurred until 2003. Therefore, the results for women born in 1974 and later were largely based on projections. The total conception rates declined in successive cohorts mostly because of a decline in the induced abortion rate. Each subsequent cohort had fewer children at younger ages, but at a later age tends to ‘catch up’ with the ‘older’ cohorts. However, part of this ‘catching up’ was explained by an increased use of ART. When the projected ART-fertility rates were excluded, cohort fertility rates already showed a slight decline for women born in the 1960s (right-hand panel of Fig. 1).

**Figure 2:**
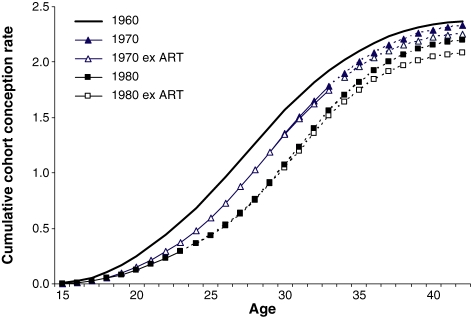
Observed and projected cumulative cohort conception rates by age with and without the use of assisted reproduction (ART) among native Danish women born in 1960, 1970, and 1980. Notes: Dotted lines show projected values. Only total conception rates are shown for the 1960 cohort as the conception rates without ART are very similar.

Figure 2 shows the observed and projected cumulative cohort total conception rates for Danish women born during 1960–1980 with and without the use of ART. Women from younger cohorts, especially those born after 1970, experienced a declining number of pregnancies, even when the use of ART was taken into account.

**Figure 3:**
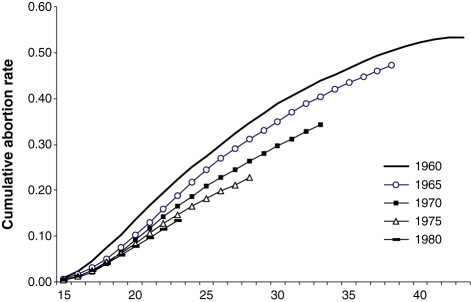
Cumulative induced abortion rates by age for selected cohorts of native Danish women born in 1960–1980.

**Figure 4:**
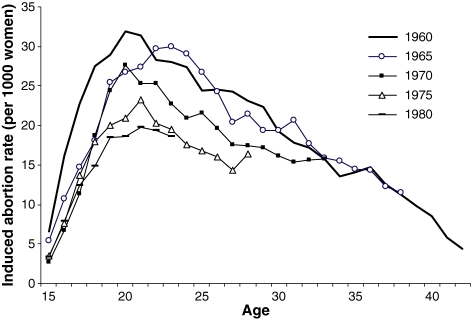
Age-specific induced abortion rates among selected birth cohorts of native Danish women born in 1960–1980.

Figure 3 shows the cohort trends in cumulative induced abortion rates. While in many countries officially registered abortions tend to underestimate the total number of abortions, in Denmark there is no reason to assume a significant undercount. There is even less reason to assume that the statistical coverage has declined over recent years and hence the observed decline is likely to be real. Induced abortions are interesting as they, to a large extent, represent unintended pregnancies. Women from younger cohorts had progressively fewer induced abortions. This trend was not linked to a postponement of births, which might be expected to lead to more induced abortions before the age of entry into motherhood. On the contrary, women had lower abortion rates at younger ages, especially at age range of 19–27 years (Fig. 3). This is clearly illustrated in Fig. 4 for the cohorts born between 1960 and 1980. Interestingly, this pattern resembles (in reverse order) the age-specific incidences in testicular cancer in birth cohorts.

**Figure 5:**
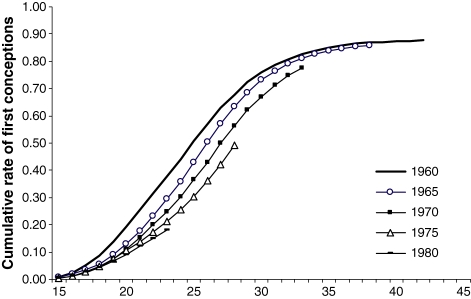
Cumulative proportion of women experiencing their first conception without the use of assisted reproduction treatment in different birth cohorts (1960–80).

Finally, Fig. 5 show the cumulative proportion of women, who by a given age experienced their first conception resulting in an induced abortion or pregnancy without the use of ART treatment. Women postponed their first conception, although they may ‘catch-up’ at later ages. However, approximately 12% of the 1960 cohort did not conceive at all and this proportion is likely to increase among women born in 1970 and later.

## Discussion

Our findings of a birth cohort-related decline in TNCR of Danish women is in line with our hypothesis that the well documented very poor semen quality in a significant proportion of young men from the general population may have a negative impact on conception rates and fertility rates among younger cohorts of Danish women. Our hypothesis is supported by the increasing and substantial use of ART, including ICSI in the management of infertility of relatively young couples in the later cohorts. In fact, women born in the mid-1970s only reach similar levels of fertility as the earlier cohorts through the use of ART (Sobotka *et al.*, 2007). We are, however, aware that male infertility alone cannot explain the observed fertility trends and that social and economical factors play a key role (Kohler *et al.*, 2006; Lutz, 2006). Women participate in the labour force; and they obtain higher levels of education, pursue non-family activities, and postpone childbearing (Sobotka, 2004) and trends in female fecundity may also contribute to the observed trend.

Beyond any doubt, the use of contraception has played a major role in the evolution of fertility and induced abortion rates we have been witnessing over the past 30–35 years. On the other hand, in a new report from the Danish National Board of Health investigating the sexuality of young Danes, it was found that almost half of all young men and one third of young women between the ages of 20 and 24 years did not consistently use contraception when having sexual intercourse with a new partner. This suggests that the younger cohorts do not use contraception more effectively that the previous cohorts (Knudsen, 2007). In addition, all contraceptive methods have significant failure rates because of various technical and psychological difficulties involved in proper use of the method in question (Fu *et al.*, 1999; Lakha & Glasier, 2006). Theoretically, the postponement of first birth observed since the early 1970s, should have resulted in more induced abortions at younger ages on account of a longer exposure to ‘accidental’ contraceptive failure. That has apparently not been the case: in contrast, the cohort abortion rates among women who have not reached the mean age at first birth – currently around 29 for native Danish women – have been steadily decreasing. One obvious reason could be that the quality and compliance of the contraceptive methods has improved over time. However, effects of improved contraceptive methods would most likely have resulted in a period effect, i.e. a decline in induced abortions for all women across all cohorts, and not in the observed birth-cohort related decline in abortions. We therefore hypothesize that the decreased conception rate among younger cohorts of women may partly be explained by poorer semen quality among their partners, who most likely belong to birth cohorts of the same age or only slightly older, and partly by a cohort effect in adapting to new contraceptive methods.

Total fertility rate, the most commonly used indicator of period fertility, is now well below the replacement level of 2.1 children per women in most European countries and parts of Asia. In many countries, the TFR is even below 1.3 children per woman (Kohler *et al.*, 2002). During the past decades, some countries, such as Russia, have had very low TFR despite high pregnancy rates. In fact, during the 1970s and 1980s, the average Russian woman had twice as many induced abortions as births (Popov & David, 1999). On the other hand, the low fertility rate in Denmark has been paralleled by a significant decline in induced abortions. The current abortions to births ratio is as low as 0.23 (2005). We therefore believe that the TNCR may be a more informative measure of the ability to conceive than the TFR which is obviously not a very sensitive measure of fecundity. Fecundity can be measured more precisely by the use of the time taken to conceive (Time To Pregnancy, TTP; see Baird *et al.*, 1986). TTP has been proven to be a valuable tool to measure fecundity; it is also well recalled (Zielhuis *et al.*, 1992; Joffe *et al.*, 1995) and related to semen quality (Bonde *et al.*, 1998a). Little is known about the true extent of, and time trend in, fecundity because population-based data are generally not available. Some population studies have reported no secular decline in fecundity (Mosher & Pratt, 1990; Templeton *et al.*, 1991; Jensen *et al.*, 2005). In Britain, a cross-sectional study showed significantly increasing fecundity over time (Joffe, 2000); and two transient dips in fertility were observed. There are, however, several pitfalls when studying TTP (Joffe, 2003; Olsen & Rachootin, 2003; Joffe *et al.*, 2005). In addition, TTP is not recorded in population-based registries so it cannot be assessed in large register studies like this.

There are reasons to suspect that semen quality among young Danish men have reached a lower threshold where fecundity and fertility of the population may be affected (Andersen *et al.*, 2000). The importance of the deterioration of semen quality for the declining fertility rates is even more important in the light of the postponement of childbearing. The average native Danish woman delivers her first child around age of 29 (own computations) and women with university education typically enter motherhood after the age of 30 (Lappegård & Rønsen, 2004). As female fecundity rapidly decreases after the age of 35 years (Baird *et al.*, 2005) and male fecundity also shows an age-related decline (De La Rochebrochard *et al.*, 2003) the time window during which couples are able to reproduce has become quite narrow.

All industrialized countries seem to experience increasing problems with male reproductive health (Andersen *et al.*, 2000; Jorgensen *et al.*, 2002; Swan *et al.*, 2003), best documented with regard to testicular cancer (Huyghe *et al.*, 2003), which seems to be a strong sensor of reproductive health of a population (Jacobsen *et al.*, 2000a,b; Skakkebaek *et al.*, 2001). In several areas of the EU, the semen quality of young males has been checked and surprisingly many young men have values below the WHO guidelines. Furthermore, testicular cancer rates have risen sharply in most industrialised countries in Europe as well as in the United States and in Australia (Huyghe *et al.*, 2003; McGlynn *et al.*, 2003). These clinical manifestations do not occur at random: they are all well documented risk factors for each other. In addition, laboratory studies of testicular specimens have demonstrated common dysgenetic changes in the testicles of men with testicular cancer, cryptorchidism, hypospadias and infertility. There is evidence that these disorders can be linked through a condition of prenatal origin, a testicular dysgenesis syndrome (TDS) (Skakkebaek *et al.*, 2001; Nistal *et al.*, 2006; Rajpert-De, 2006). The aetiology of this syndrome, which apparently is more common among the younger birth cohorts of men, is unknown, but the sharp increase in male reproductive disorders over one or two generations suggests that environmental factors must play an important role. Interestingly, animal models have recently been developed, showing that a TDS-like syndrome can be generated in rats exposed to endocrine disrupters, including phthalates (Andrade *et al.*, 2006; Mahood *et al.*, 2006). Several European, North American and Asian research programs have been developed to explore the possible connections between exposure to endocrine disrupters and human male reproductive problems (Toppari *et al.*, 1996). Few studies have recently appeared linking testicular cancer to maternal exposure to polychlorinated biphenyls (PCBs) and brominated flame retardants; phthalates and organochlorine pesticides to developmental problems in genitalia of newborn boys (Swan *et al.*, 2005; Damgaard *et al.*, 2006; Hardell *et al.*, 2006; Main *et al.*, 2006).

There is further support for this hypothesis from the non-biological field of changing fertility intentions and ideals. A series of Eurobarometer studies (Testa, 2007) showed that the mean intended family size expressed in these surveys by young Danish women has been rather stable over time and even slightly increased between 2001 and 2006, reaching 2.24 children per woman. Although these data have not been directly matched with the cohorts studied here they also tend to support the general view that the decline in fertility rates among the younger cohorts may have been partly involuntary. We also cannot exclude the possibility that decline in female fecundity because of, for example, increasing prevalence of sexually transmitted diseases may contribute to the observed decline in TNCR, although no data are available to address this topic.

There are limitations to our register-based study. It has an advantage of relying on valid and practically complete national data of the Danish Induced Abortion Registry and the Danish Medical Birth Registry. The ART Registry, which has only recently been established, is only complete with regard to IVF and ICSI since 1996. It also includes pregnancies after IUI performed at public clinics. However, conceptions after IUI performed in private clinics are not always included in this registry, which will lead to an overestimation of the TNCR. ART treatment for the first child with the current partner among resident women in Denmark is free of charge, a fact which may partly contribute to the high rates of ART pregnancies observed in Denmark. In addition, a small fraction of couples conceiving after the use of ART treatment may actually have been able to conceive ‘naturally’ if the treatment had not been initiated (Leridon, 2004). This might have overestimated the effect of ART slightly. Obviously, there are also limitations to the projections we made, but as they are based on rather ‘conservative’ estimates of future trends, we believe they do not overestimate the pace of decline in TNCR and the importance of ART in sustaining fertility rates in Denmark.

## Conclusions and perspectives

The low fertility rates we have witnessed in Denmark during recent decades are similar to those seen in many other industrialized countries. They have been associated with falling induced abortion rates, particularly among the younger birth cohorts. Although a lower rate of induced abortions in itself is a positive development and often seen as a product of improved public health education programs resulting in more effective contraception, it is pertinent to study seriously the possible role of decreased fecundity for this development in human reproduction. While social and economic trends influencing fertility are difficult to reverse this is even more the case for biological changes that evolve along cohort lines. If decreased fecundity because of progressively poorer semen quality among younger cohorts of otherwise normal men contributes to the observed decline in conception rates we may expect very serious implications for reproductive health in the future. The current widespread use of ART in Denmark may in fact be a sign of such an emerging public health problem. Hitherto, subfecundity has mainly been considered an individual problem, but if it further increases in prevalence, it may well turn into a major public burden, with profound effects on our societies. In addition, as women further postpone childbearing they are increasingly relying on ART treatment and many may therefore wait too long for realising their fertility intentions. It is relatively unknown by the general public that the ART success rates are limited among women in the late stage of their reproductive life, especially above the age of 40 years. The wider availability of ART may create a false confidence among many couples that births can be postponed towards the limits of biological fecundity (Grant *et al.*, 2006).

As the decline in conception rates, as well as the adverse trends in some markers of reproductive health, such as testicular cancer, has occurred over a relatively short period we should consider environmental exposure, including lifestyle, as determinants. The good news would be that removal of such environmental factors, once identified, should also eventually remove the problem provided that there are no transgenerational effects. The bad news is that we may not see an improvement in human reproductive function until one generation later. Recent progress in human and animal research suggests that exposure in utero and early life to environmental factors plays an important role for programming adult gonadal function. Therefore, public health initiatives to improve reproductive health may not yield the full results until some 30 years after the specific problems have been identified and corresponding action is taken.

## Contributors

The hypothesis was generated by NES and TKJ after discussions with WL. TKJ, NES and ATP obtained the data; MAH, TKJ and ATP did data cleaning. TS did the analyses and projections. All authors participated in the interpretation of the data analysis. TKJ and NES drafted the paper. All authors contributed to the editing and final adjustment of the paper.

## Funding

This study was funded by Danish Research Council (grant no 95-103-21-990), the European Commission under the EDEN consortium (contract no. QLK4-CT-2002-00603) and Danish Cancer Society. None of the funding sources had any involvement in the study and analysis.

## Panel discussion

### K.M. Grigor

There is a decline in the total number of sperm in the ejaculate, but in normal semen only about 9% of spermatozoa appear normal morphologically. Is this proportion of normal spermatozoa constant with declining total sperm count, or does the ratio of normal sperm decrease with the fall in total sperm count?

### T.K. Jensen

This point will be addressed in a later paper by Dr N Jørgensen. In our study, the young man with a low sperm count also had a relatively low number of normal sperm, and the proportion of normal sperm was lower than in a previous American study published. It is therefore probable that poorer morphology in addition to low sperm count contributes to reduced fertility.

### P. Grandjean

You indicated optimistically that removal of environmental hazards would improve fertility. However, poor semen quality may be caused by environmentally induced, but heritable, epigenetic changes. Assisted reproductive technology (ART) may contribute to the passing on of infertility to the next generation, and the ART itself may cause adverse effects on the progeny. Also, fertility treatment may impact on fertility of the progeny.

### T.K. Jensen

I was perhaps too optimistic in suggesting that improvements in the environment would be the answer to declining fertility. Logically, progeny cannot “inherit” sterility, but with ART, including intracytoplasmic sperm injection (ICSI) where only one sperm is required to inject into the egg, perhaps subfertility could be inherited.

### G. Bittner

It is possible that a decline in female reproductive health also contributes to the trend of reduced fertility. This exists although it is much more difficult to measure in comparison to the simple method of counting semen samples to assess male fertility. You have access to demographic data which could be used to compare the fertility rates of Danish male/Danish female couples with: (a) Danish male/non-Danish female couples (to assess the relative importance of Danish male fertility); and (b) non-Danish male/Danish female couples (to assess the relative importance of Danish female fertility).

### T. Sobotka

Standard demographic surveys in many countries ask women if they had wished another child, and the reasons for not having another child, including infertility. It is possible to track women wanting another child and to ask them the reasons for not having a recorded delivery for the subsequent 3–5 years. No such survey has been performed in Denmark. It is difficult to draw conclusions from comparing fertility rates of mixed native and non-native Danish couples because immigrants come from diverse populations with different fertility desires in comparison to the native Danish population. The average non-European immigrants come from high fertility regions of the world and desire more children.

### N.E. Skakkebæk

The quality of female fertility must also be considered but there are good reasons to suggest that the problem of increasing infertility is partly due to males. There are an increasing number of males attending infertility clinics for assisted reproduction (ART) using intracytoplasmic sperm injection (ICSI), which is a treatment for male infertility.

### M. Bang

We have data on a cohort of women born in Copenhagen 1959–60, treated with hormones during pregnancy: a study of their fertility would be useful. We must also consider the availability of services including day care, and social services with support for families, on birth rates. This has had a large impact on the number of children of families in Eastern European countries after 1989. Fertility rates fell in Southern Europe due to postponement of childbearing when many more women entered the workforce, and a similar effect was seen in Denmark in the late 1970s.

### T. Sobotka

These data are more suitable for a comparison of international fertility rates.

### G. Potashnik

Dr Skakkebæk asked how we can learn from animal experiments to understand the human situation and Dr Skakkebæk's group has published many papers on animal experiments. It is well known in toxicology that we must be very careful when extrapolating animal results to humans because many different factors are different. Clinicians must be aware of the ethical aspects of treating infertility including assisted reproductive technology (ART) which has been used to produce 100,000s of babies. In many cases, the outcome of pregnancy has been prematurity, multiple births and congenital malformations. The treatment of infertility may not be the best answer for the future of the human race.

### N.E. Skakkebæk

The consequences of ART have not been fully discussed at this Workshop. Much work is still required to study the babies born after all forms of fertility treatment.

### S. Swan

The outcome of assisted pregnancies must be assessed including the overall cost which involves the financial implications, the burden to society and the emotional cost to the parents.

### R.J. Aitken

The overall impact of ART to society will increase as we look into the future. If we are required to use ART to maintain the population at the current level, this will have long term genetic consequences. There will be an increase in the number of infertility genes in the population because we are no longer selecting for high fecundity.

### W. Lutz

There is a suggestion of a widespread infertility epidemic in industrialised countries, but from a demographic point of view it is too early to be certain and it depends on the definition. It is unclear if the present level of infertility will be an important factor for future fertility. Demographers assume that the birth rate is controlled by decision making and social actions to conform to social norms, as a result of use of contraception which can alter both male and female fecundity.

The challenge for research workers is to decide which data must be assessed to decide if low birth rate is a consequence of infertility. Papers at this Workshop by Dr TK Jensen and Dr T Sobotka indicate that the trend in induced abortion is an important factor in birth rate, but accurate data on use of contraception is lacking. A series of surveys (WFS, FFS, GGS) has collected data on family planning methods used, and planning status of pregnancies by order of birth. Mothers were asked if their pregnancy was planned or not, and a trend in unplanned pregnancies has emerged. In Austria, 40% of first births were unplanned, but this was mainly due to timing failure whereby the baby arrived too early but was not unwanted. More than 80% of 2^nd^ births were planned but 3^rd^ and 4^th^ births were increasingly unplanned presumably as a result of inefficient contraception. A more comprehensive overview could emerge by performing similar surveys in many more countries.

### T. Sobotka

Surprisingly little is known to what extent the low birth rate is due to infertility problems, although a number of surveys have looked at the proportion of women who are infertile at different ages. Tentative results based on the surveys of fertility intentions suggest that infertility is not the most important factor, but social reasons are the main cause of low birth rate linked to the preference for a small family size. The recent Eurobarometer 2006 survey reported by Maria Rita Testa asked women how many children they had intended to have when they were 20 years old, and why this number was not achieved. Although health related reasons in the couple were cited most frequently by women (they were mentioned by 17% of female respondents) it is unclear what fraction of these health reasons refer to infertility problems. Besides health reasons, two most important reasons cited given for not fulfilling their fertility intensions were lack of a proper and supportive partner, and change of priorities. Of less importance were economic reasons including cost of housing, and difficulty in employment (M Rita Testa. 2007. “Childbearing preferences and family issues in Europe: evidence from the Eurobarometer 2006 Survey” Forthcoming in *Vienna Yearbook of Population Research 2007)*.

### S. Swan

The sex ratio is an important factor in population studies and there is a general trend in many countries towards a lower proportion of boys. Is this considered in predictions of future populations?

### W. Lutz

Sex ratio is a controversial topic especially in China and India where traditionally there has been a strong preference for male children. In some countries prenatal sex screening is illegal in order to prevent sex-specific abortion. In China, there is a very high M:F ratio of 114–116:100 resulting in distortion of the balance in later adult life and social unrest because the number of partners does not match. This is also affected by the differential migration of the sexes to cities. There is a changing outlook in cities in China because couples assume that they will be looked after better by daughters in later life, and the prospective usefulness of female babies may have an effect on the sex ratio.

### A.-M. Andersson

It has been suggested that the decrease in induced abortions may be due to better use of contraceptives. Recently the Danish media has highlighted an increase in sexually transmitted diseases (STDs) indicating a deficient use of contraceptives, or using contraceptive methods which do not protect against STDs. Information on the incidence of STDs and the type of contraceptive used may indicate if the decrease in induced abortion rates is due to less unwanted pregnancies or less unprotected intercourses.

### M. Bang

We must discuss the most productive approach to develop a strategy to cope with the problems associated with endocrine disruption including possible preventive measures. Between 1997 and 2004 in Denmark there has been a three-fold increase in the number of unrelated disease groups caused by endocrine disrupters including brain defects and allergies in addition to abnormalities in the reproductive tract. There has been some skepticism in the broader medical community about the significance of endocrine disruption, but the economic consequences of brain effects must be considered including the social costs of childhood defects including autism and ADHD. There has been a recent decrease in the level of average intelligence in conscripts in Norway and Denmark after a period of 50 years of constant rise, which may be due to environmental influences.

### N.E. Skakkebæk

I agree that the medical profession appears not to be addressing these problems adequately. This is surprising considering that doctors are trained to detect early signs of disease so that treatment can be instigated to avoid catastrophe. However, we are less inclined to tackle changes in disease processes in society as a whole rather than individuals.

### S. Swan

The time to pregnancy described by Dr TK Jensen is an important factor in fertility rates.

### T.K. Jensen

Easy ways to measure fertility is to examine semen samples in males, and to ask couples the time taken to become pregnant. Questions in questionnaires included: Was the pregnancy planned? How long did you take to conceive? Did you use contraceptives? The time to pregnancy (TTP) data have been published by Mike Joffe (Am. J. Epidemiol.). A recent study in the Faroe Islands had to be anonymous because in a small community it was difficult to ask how often they had sexual intercourse. However, there was good recall, and 90% of the appropriate respondents could remember the last 40 years. Such surveys give a good measure of population fecundity, and unsuccessful attempts can be assessed. We had two questionnaires, one for women who had children and one for those who were never pregnant including a question on whether they had intended to become pregnant or not, and if so, for how long and why did they stop.
